# Optimising cool-water injections to reduce thermal stress on coral reefs of the Great Barrier Reef

**DOI:** 10.1371/journal.pone.0239978

**Published:** 2020-10-20

**Authors:** Mark Edward Baird, Rebecca Green, Ryan Lowe, Mathieu Mongin, Elodie Bougeot

**Affiliations:** 1 CSIRO Oceans and Atmosphere, Hobart, Tasmania, Australia; 2 UWA Oceans Institute, Ocean Graduate School and ARC Centre of Excellence for Coral Reef Studies, University of Western Australia, Perth, Western Australia, Australia; 3 École Nationale Supérieure d’Ingénieurs de Limoges, Limoges, France; Living Oceans Foundation, TAIWAN

## Abstract

Coral bleaching driven by ocean warming is one of the most visible ecological impacts of climate change and perhaps the greatest threat to the persistence of reefs in the coming decades. In the absence of returning atmospheric greenhouse gas concentrations to those compatible with ocean temperatures below the mass coral bleaching temperature thresholds, the most straightforward means to reduce thermal-stress induced bleaching is to cool water at the seabed. The feasibility of reducing the seabed temperature through cool-water injections is considered first by analysing the feasibility of doing so on 19 reefs with differing physical environments using a simple residence time metric in 200 m resolution hydrodynamic model configurations. We then concentrate on the reefs around Lizard Island, the most promising candidate of the 19 locations, and develop a 40 m hydrodynamic model to investigate the effect of the injection of cool water at differing volumetric rates. Injecting 27°C seawater at a rate of 5 m^3^ s^−1^ at 4 sites in early 2017 cooled 97 ha of the reef by 0.15°C or more. The power required to pump 5 m^3^ s^−1^ through a set of pipes over a distance of 3 km from a nearby channel is ∼466 kW. This power applied at 4 sites for 3 months achieves a 2 Degree Heating Weeks (DHWs) reduction on 97 ha of reef. A more precise energy costing will require further expert engineering design of the pumping equipment and energy sources. Even for the most physically favourable reefs, cool-water transported through pipes and injected at a reef site is energy expensive and cannot be scaled up to any meaningful fraction of the 3,100 reefs of the GBR. Should priority be given to reducing thermal stress on one or a few high value reefs, this paper provides a framework to identify the most promising sites.

## Introduction

The greatest threat to coral reefs on global scales is coral bleaching [1, 2]. Coral bleaching is the expulsion of unicellular zooxanthallae symbionts from the coral host, often leading to mortality. The main drivers of coral bleaching are thermal and light stress [3]. The mechanism by which temperature and light drives bleaching is through the build-up of reactive oxygen stress inside the coral host due to inactivation of the zooxanthallae photosystem at anomalously-high summertime temperatures [4, 5]. As a result of the direct dependence of bleaching on environmental temperature and light levels, researchers have advocated interventions to reduce light reaching corals and/or to cool the water surrounding corals [6–8].

The different schemes that have been proposed to reduce the temperature of water above the corals can be broadly grouped [7] into those that reduce solar radiation over a large area through shading or cloud brightening [9], and those that cool the water directly through processes such as enhanced vertical mixing [10]. This paper investigates cool-water injection delivered to the site of corals through pipes, similar to that trialed by the Climate Foundation near Tutuila Airport, American Samoa [7]. In contrast to schemes that encourage mixing, the delivery of cool water through pipes ensures that the water initially arrives at the required site without warming through dilution. But delivery by pipes comes at the large cost of the energy of pumping.

Numerical models can be used to investigate the feasibility and cost of environmental interventions before trials are undertaken. This is a necessity for global scale geo-engineering proposals [9, 11] which would never be considered without a highly credible model representation of the impact of the intervention. Numerical modelling is equally important for more localised interventions [12] in order to obtain the most cost effective application of the intervention.

In this study, we apply a suite of hydrodynamic models to investigate the effectiveness of cool-water injections to mitigate the effects of thermal stress on coral reefs that are part of the Great Barrier Reef (GBR). The temperature of water over the reef on any particular day depends on how large scale oceanographic and meteorological processes interact with the morphology of individual reefs [13, 14]. Thus we use the 1 km resolution eReefs hydrodynamic model [15] to consider remote forcing of the reef. We then use nineteen 200 m resolution hydrodynamic models, configured for the summer of 2017 when the GBR bleached severely, to determine the reef with the most favourable hydrodynamic environment for cool-water injection. For the most suitable reef, we develop a 40 m resolution unstructured hydrodynamic model to capture the impact of the optimally-located cool-water injection on seabed temperature on the reef. Finally, as the optimal reef has a nearby 40 m deep channel containing cooler water, we estimate the energy costs associated with pumping this cool water to the injection sites. By this means we both give cool-water injection the best chance to succeed in reducing thermal stress by choosing a highly favourable location and provide preliminary calculations to guide how the approach might be optimised.

## Materials and methods

To consider a local cool-water injection, we assume that the water offshore of the reef on which our intervention site is located remains at the ambient temperature in the absence of intervention. That is, the water flowing onto the chosen reef from deeper inter-reef areas is the same temperature as if no intervention existed. Thus to cool water at a particular site we need to cool the large volumes of water that move across the site from outside the area of influence of the intervention. Most reefs of the GBR are exposed to significant tidally-driven circulation [16], so cooling the water moving across the site becomes the primary challenge.

This study used a curvilinear hydrodynamic model configured for the GBR with ∼200 m nests around 19 individual reefs to select the most hydrodynamically-suitable reef; and a newly-configured high resolution unstructured hydrodynamic model for the modelling of cool-water injection on the chosen reef. This combination of hydrodynamic modelling approaches allowed us to optimise cool-water injection based on the range of scales (global to meters) of processes that regulate the temperature of reef waters.

### Nested reef-scale models for optimal reef selection

A large, multi-agency collaboration has developed the eReefs coupled hydrodynamic, sediment and biogeochemical model that simulates at multiple scales the environmental conditions of the GBR [17, 18]. The project provides ∼1 and ∼4 km resolution hindcast and near real time simulations of hydrodynamic and biogeochemical quantities. The models provides skilful predictions of temperature across the entire GBR [15, 19]. The 1 km resolution model has run from Dec 1, 2014 to present, and we use the output from 1 Dec 2016—31 Mar 2017 during the severe bleaching of 2017 [20].

The eReefs project includes semi-automated model generation that allows high-resolution models to be nested within the 1 km regional hindcast (RECOM—RElocatable Coastal Ocean Model, [5, 19]). We created reef scale models around ∼50 reefs, 19 of which are shown in this paper. Each nested model uses hydrodynamic boundary conditions provided by the 1 km model (designated GBR1_H2p0). The boundary conditions for temperature and salinity are formulated using the advection scheme used within the model domain itself [21]. This consistency of boundary and advection schemes ensures diffusion and dispersion errors are minimised.

The effectiveness of cool-water injections is determined primarily by the hydrodynamic processes (such as tidal and wind driven flows) that transport the injected water off the reef. To provide a spatially-resolved quantification of the sum of these processes we have calculated a residence time metric using the hydrodynamic simulations. “Reef age” is a measure of the time [in days] a parcel of water has spent over a reef, and varies in space and time. To calculate “reef age” requires consideration of the transport of water over the reef, as quantified by the same set of advection-diffusion equations used for conservative tracers in the hydrodynamic model. Furthermore, “reef age” has an accumulation term over time for water parcels above the reef. Thus the equation for “reef age”, *τ*, becomes:

$$\begin{array}{c} \frac{\partial \tau }{\partial t}+u•\nabla \tau =\nabla •(\nabla \;K)\tau +\;\Phi ,\;\Phi =1\;(above\;reef),\;\Phi =0\;(open\;ocean)\; \end{array}$$ (1)

where ∇ = ∂/∂*x* + ∂/∂*y* + ∂/∂*z* is the gradient operator, *u* is the velocity vector, *K* is the spatially and temporally- resolved diffusion coefficient, and Φ is the source term defined as 1 d d^−1^ above the reef and zero in the open ocean. The four terms in Eq 1, from left to right, are the local rate of change, advection, diffusion and accumulation. For the reef age tracer we have defined the spatial extent of the reef by model cells with a bottom depth shallower than 10 m. Further explanation of age tracers is available for marine modelling [22, 23] and reef modelling [12].

### Modelling cool-water injection

In order to model hydrodynamic processes at close to the scale of a plume from an pipe outlet, the Delft3D Flexible Mesh (Delft3D FM) modelling suite was used to nest a finer resolution model into the 1 km eReefs hydrodynamic model simulation (GBR1_H2p0). Delft3D FM is an open source unstructured grid model maintained by Deltares (http://oss.deltares.nl/web/delft3dfm) that solves the 2D and 3D shallow water equations using finite volume schemes [24], representing a major redevelopment of the widely-used Delft3D (structured grid) model [25]. The model domain covered ∼7 × 8 km, and was mapped onto a grid composed of triangular cells, with a horizontal resolution varying between 40–50 m, and 4 sigma layers in the vertical. High resolution (30 m) bathymetry data compiled from LiDAR, multibeam and satellite data [26] were interpolated onto the grid.

Boundary conditions were defined using eReefs GBR1_H2p0 output, and tidal constituents obtained from the TPXO7.2 global tide solution [27]. The model ran over a full spring-neap cycle in January 2017, during which time meteorological conditions (solar radiation, humidity, air temperature, cloud coverage and wind) were prescribed using the same BoM ACCESS-R products used by the 1 km eReefs model. The components of the Deflt3D hydrodynamic model setup that rely on the outcome of the reef selection are given in the Results section.

### Energy costs of injections

The water for injection will need to be transported both horizontally and vertically from a location that contains cooler water. In order to approximate the costs of pumping cool water to the reef we have undertaken some simple engineering calculations for pipe flow, following the well-known Moody diagram, as outlined in [29] and [30] and summarized in Table 1. The large volumes of water (up to 10 m^3^ s^−1^ to each site) determine that the most likely design would involve multiple pipes to each site.

**Table 1:** Energy calculations for turbulent flow through a circular pipe. In these equations, the volumetric flow, *V*, is for each pipe. Thus, for a required supply of 5 m^3^ s^−1^ through 4 pipes, *V* = 1.25 m^3^ s^−1^.

*V*	volume transport per pipe [m^3^ s^−1^]
*D*	pipe diameter [m]
*n*	number of pipes
*L*	pipe length = 3000 m
*h*	depth of cool water = 40 m
*ρ*	seawater density (at 35 S, 27°C) = 1022.72 kg m^−3^
*g*	acceleration due to gravity = 9.81 m s^−2^
*μ*	kinematic viscosity = 1.08 × 10^−3^ kg s^−1^ m^−1^
*c*_*p*_	specific heat capacity of seawater = 4.186 J g^−1^°C^−1^
*A*	cross-sectional area of pipe, *π*(*D*/2)^2^ [m^2^]
*U*	pipe velocity, *V*/*A* [m s^−1^]
*ϵ*	wall roughness = 2 mm
Re	Reynolds number (turbulence measure for a pipe), *ρUD*/*μ* [unitless]
*f*	rough pipe friction factor [28], $1/\sqrt{f}=-1.8\;{\text{log}}_{10}{\left(\left(\epsilon /D\right)/3.75\right)}^{1.11}+6.9/\text{Re}$
Δ*p*	pressure drop along pipe *L* m long, *fρU*^2^ *L*/2*D* [kg m^−1^ s^−2^]
*P*_*f*_	power to overcome friction, Δ*pV* [W]
*P*_*m*_	power to accelerate flow, 0.5 *ρVU*^2^ [W]
*P*_*l*_	power to lift *h* meters, *gV*(*ρ*_35,27_ − *ρ*_*w*_)*h* [W]
*P*_*c*_	power to cool water by Δ*T*, *c*_*p*_ *ρV* Δ*T* [W]
*φ*	pump efficiency = 80%

Energy costs include friction loss due to flow through the pipe, as well as the energy to lift the water to near the surface and to accelerate the water from rest at the inlet to the flow velocity required in the pipes. The major assumptions of the calculations include: (1) the flow is constant through the pipes with a constant wall roughness; (2) there is no energy loss due to potential bends in the pipe; (3) the pipe is submerged at both ends, so lift is based on the density difference between the inlet and outlet, rather than the density difference between the inlet and the atmosphere; (4) the pipes are of equal dimensions and equal flow; and (5) there is no gain in thermal energy as the cool water moves through the pipe. These calculations are sufficient to indicate the approximate energy costs of pumping. A multitude of other factors such as increased friction due to biofouling are likely to be important in a more detailed analysis of cost.

## Results and discussion

### Nested reef-scale models for optimal reef selection

**Fig 1:**
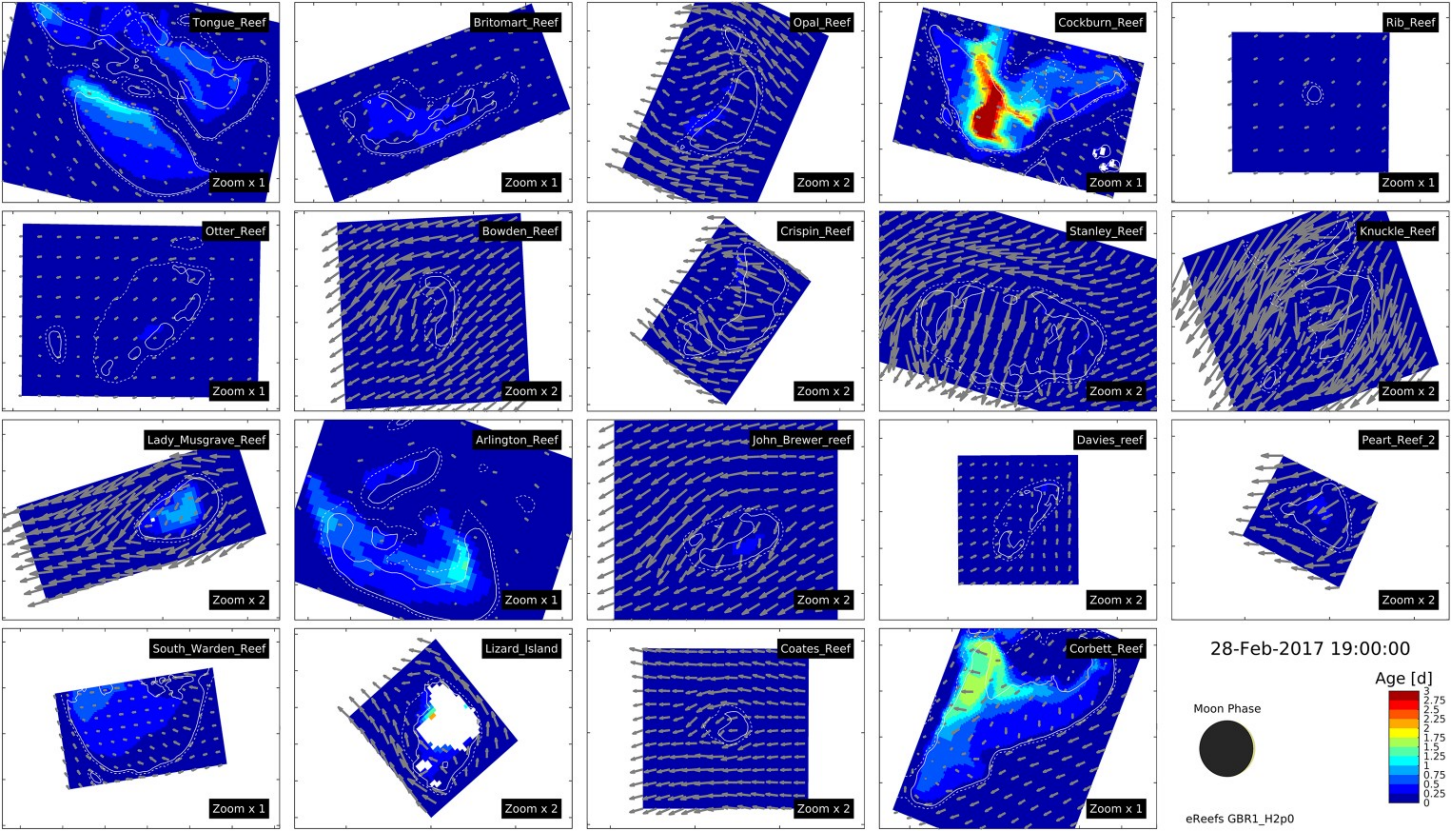
Reef age in surface waters. Reef age (colour) of the surface water on 19 reefs of the GBR at 19:00 28 Feb 2017 (UTC +10) with surface currents shown with grey quivers. To account for different sizes, the smallest reefs are zoomed by twice the largest reefs. The moon phase is shown schematically in the right bottom to emphasise the dependence of reef age on the spring-neap cycle, along with the date and time of the image. The solid and dashed white lines are the 10 and 20 m depth contours. The length of the quiver is the distance water moves in 60 minutes. An animation from 30 Jan—28 Feb 2017 appears in S1 Fig.

**Fig 2:**
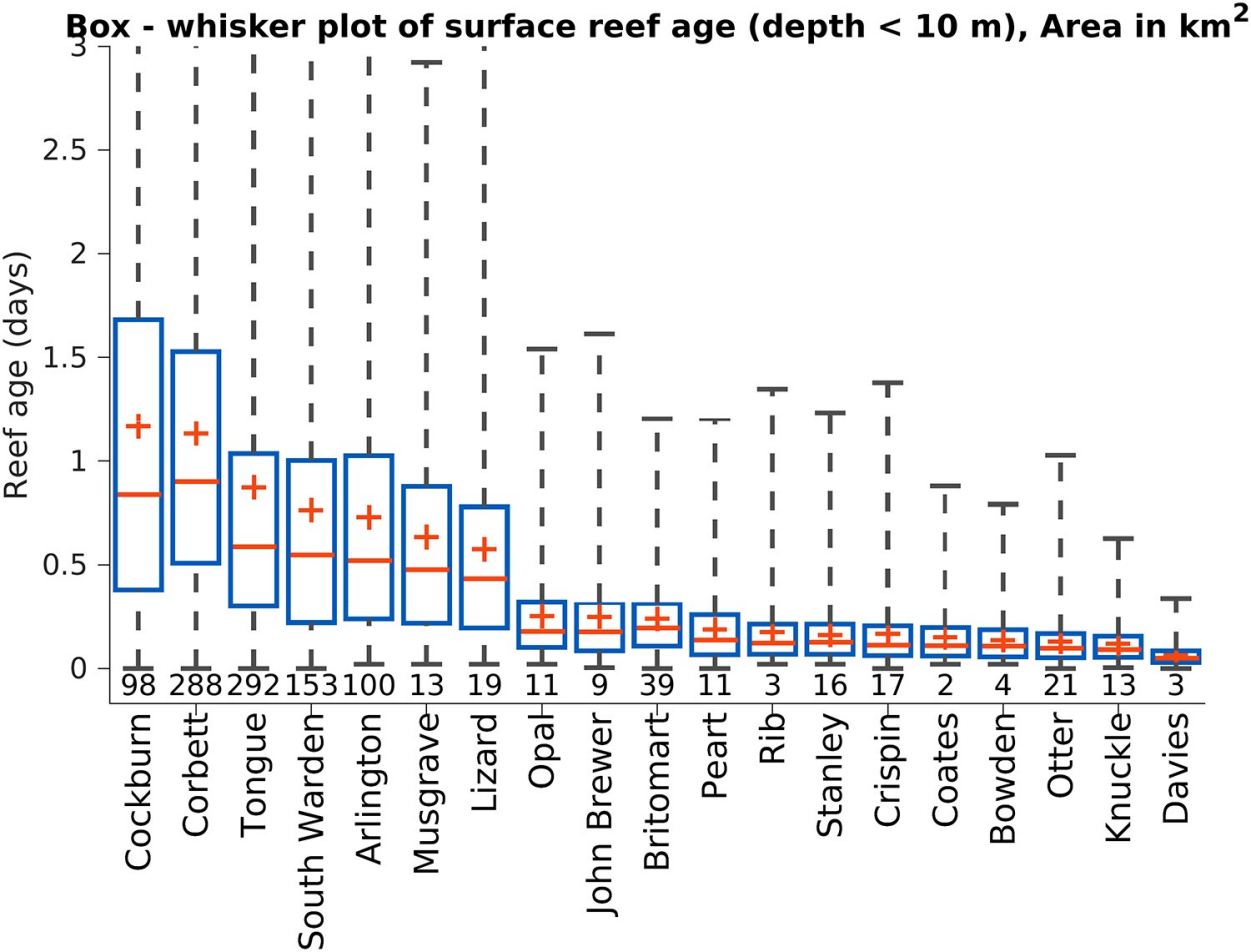
Distribution of reef age in surface waters. Statistics of surface reef age in shallow waters (depth < 10 m) of 19 reefs of the GBR, sampled every three hours in each shallow model grid cell between 30 Jan 2017—28 Feb 2017. The number at the base of each plot is the surface area (km^2^) of the reef with a depth less than 10 m. Blue boxes extend from the 1st to 3rd quartiles, red crosses are mean, red lines the median and the whiskers show the range.

In choosing an optimal reef for cool-water injection, we have used reef age, *τ*, that provides a spatially-resolved quantification of the time water has spent within a reef. The mean surface reef age of 19 reefs are shown in Fig 1 and its statistical distribution quantified in Fig 2. The longest reef ages are found on Cockburn, Corbett, Tongue, Arlington and South Warden. Each of these reefs are large (>98 km^2^ for depth < 10 m), allowing the water to reside on top of the reef for more than one tidal oscillation. In these reefs, reef age reflects the residual flows over multiple tidal cycles. On smaller reefs such as Davies and John Brewer Reefs, reef age can be less than 6 hours as each tidal phase replaces the water above the reef.

In addition to reef size, geometric alignment of the tidal ellipse with the reef shape is also important—if the long axis of the tidal ellipse aligns with the longest dimension of the reef this also adds to the mean reef age of the overlying water. While Cockburn, Corbett, Tongue and Arlington have the longest reef ages, the regions with highest reef age on these reefs are generally in water overlying sand-dominated reef flats with low coral cover. This is not coincidental. Water that has circulated over shallow nutrient-limited benthic communities quickly becomes deplete in nutrients, and thus can not support the diverse coral communities seen on reef slopes and reef crests exposed to greater dissolved and organic nutrients from off the reef [31].

Of the 19 reefs (Fig 2), the two reefs that have the longest reef age over coral communities are the reef flats of Lizard Island and Lady Musgrave Island. Lizard Island contains a research station and is in the northern GBR that suffered from severe bleaching in 2016/17 [2] while Lady Musgrave Island is in the southern GBR, suffered less thermal stress in the last decade, and has minimal development. Thus, for this study we will concentrate on Lizard Island. Based on this analysis, we conclude that Lizard Island reef flat is in the top 10% of reefs for the effectiveness of cool-water injection. Coincidentally a 40 m deep channel, which connects at depth with cooler slope waters that lie a further 10 km to the east, is found 3 km to south of the Lizard Island reef flat. Thus the Lizard Island reef flat, above all others, warrants the further investigation for cool-water injection.

### Impact of cool-water injection

**Fig 3:**
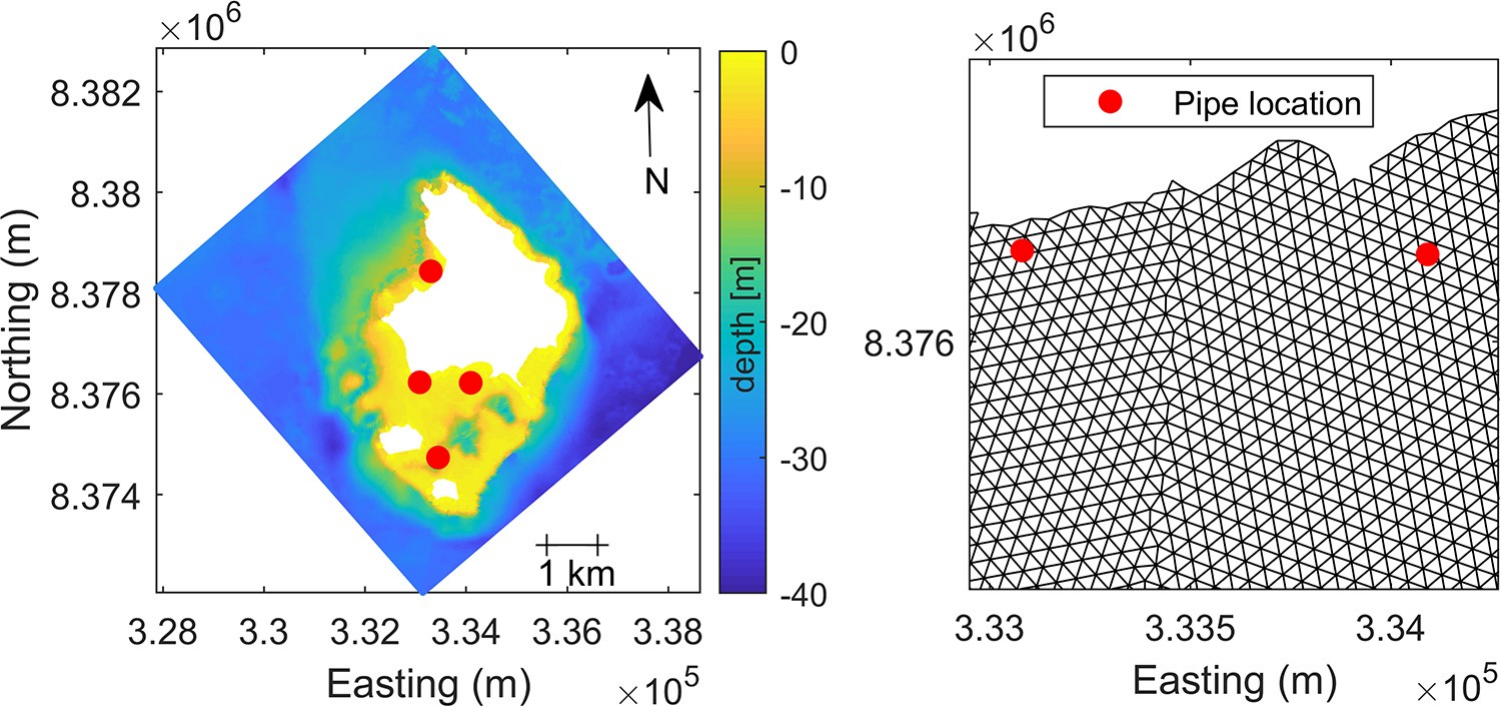
Cool-water injection hydrodynamic grid. Delft 3D FM model. (a) model domain with interpolated bathymetry from [26], location of cool-water injections shown in red; white areas inside model domain indicate dry land. (b) zoom-in on grid mesh south of main island.

The 40 m resolution, unstructured grid hydrodynamic model developed for capturing the injection plume is shown in Fig 3. To investigate the effect of pipes delivering cool water to reduce thermal stress on the reef, we first ran a control (or no injection) simulation, which is our best estimate of the conditions through the two weeks. We then ran additional simulations in which we injected 27°C water. This temperature is based on the mean observed temperature in a nearby 40 m deep channel during the simulation period, and is approximately 1°C cooler than the average temperature on the reef flat without intervention. The difference in hydrodynamic properties between the control and injection scenarios is taken to be the effect of the cool-water injection.

For this initial investigation, four pipe locations in the reef waters surrounding Lizard Island were chosen at depths between 2.3–16.6 m (Fig 3). These locations were chosen by identifying regions of the highest reef age of surface water (Fig 1), i.e. sites where the cooler water would not be advected off the reef rapidly, and therefore more likely to successfully reduce the reef temperature. Different pipe locations would produce spatially variable temperature reductions, and thus careful optimisation would be necessary before implementation to target sites with the maximum sustained decrease in temperature.

Different injection scenarios (from 2 to 50 m^3^ s^−1^) of 27°C water were trialled at varying time intervals. Both periodic (e.g. when solar radiation was strongest between 11:00–14:00) and continuous (e.g. over the two week simulation period) injections of cool water were tested. For simplicity, we present results from three simulations with continuous, feasible flow rates (2, 5, 10 m^3^ s^−1^) at each of the sites. For this initial investigation, four pipe locations in the reef waters surrounding Lizard Island were chosen, at depths between 2.3–16.6 m (Fig 3). Periodic injections were considered less effective, due to rapid mixing and dilution with warm surrounding waters, and the dependence on the tidal phase. For the maximum 10 *m*^3^ *s*^−1^ injection rate, the majority of the reef experienced a mean seabed temperature reduction of less than 0.05°C under periodic injections (not shown), thus the periodic injection is unlikely to reduce thermal stress and mitigate coral bleaching.

For the simulations analysed in this paper, the source water was introduced continuously over the simulation period from outside the model domain into the bottom layer of the model. The average temperature difference with an injection of cool water was calculated over the spring-neap cycle, as well as the area of reef where temperature was reduced.

**Fig 4:**
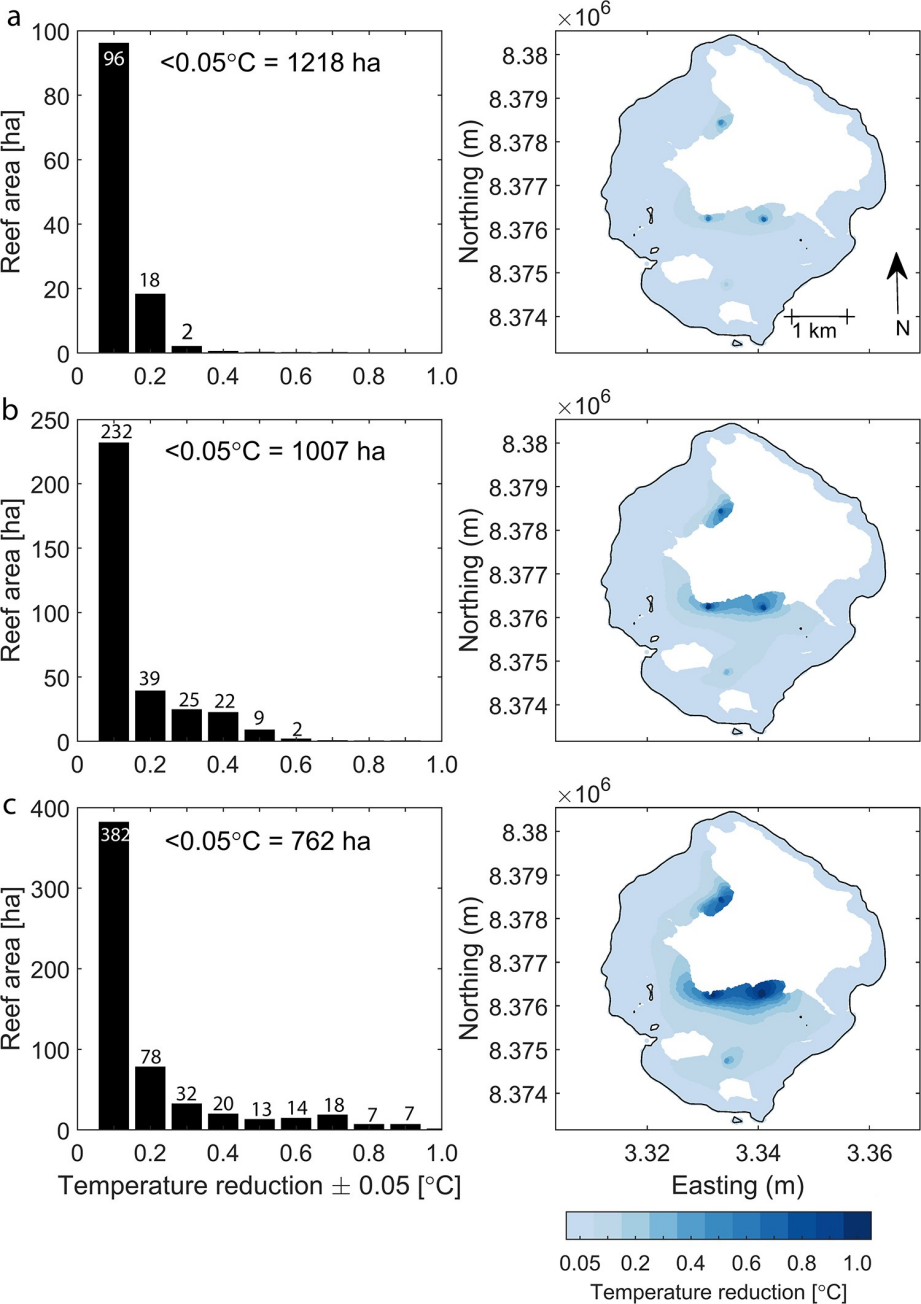
Temperature reductions on Lizard Island. Mean seabed temperature reduction for (a) 2, (b) 5 and (c) 10 *m*^3^ *s*^−1^ continuous injection of 27°C water averaged over the reef for a spring-neap cycle in January 2017. Left column: Histogram of total reef area (y axis) subject to specific temperature reductions (x axis). Only values where area > 1 ha are displayed. Total reef area (1334 ha) was defined as the area where depth < 20 m. Right column: Spatial distribution of seabed temperature reductions.

Results from three continuous discharge scenarios (2, 5, 10 m^3^ s^−1^ at each site) are shown in Fig 4. The greater discharges correspond to both a larger temperature reduction over the whole reef, and a bigger area of reef influenced (Fig 4, bottom row versus top row). There was minimal interaction between the northeastern injection site, and the three injection sites in the lagoon. For the 2 m^3^ s^−1^ scenario, the area of reef where the temperature is reduced by > 0.05°C is 117 ha, and > 0.25°C is 3 ha (Fig 4d). In comparison, for the 10 m^3^ s^−1^ scenario, the area of reef where the temperature reduction is > 0.05°C is 571 ha, and for a reduction > 0.25°C is 111 ha (Fig 4f).

### Energy costs of injections

The energy losses due to friction (*P*_*f*_) dominates over that for momentum losses (*P*_*m*_) or to lift the water (*P*_*l*_) (Table 2). These friction and momentum terms are proportional to *U* and *U*^2^ respectively, which itself is inversely proportional to the pipe diameter. That is, when the pipe diameter is reduced for a given volumetric flow rate, the costs increase to an exponent greater than 1. However, large diameter pipes are expensive, may affect the aesthetics of the reef, and will impact on coral communities in their path. Thus we have calculated losses for just 1 m diameter pipes. Therefore to alter the flow in pipes we have changed the number of pipes at each site over which the flow is divided.

**Table 2:** Results of energy calculations. Calculated pipe flow rate, friction loss, momentum loss, lift loss, total loss and cooling rate for one site in each of the three cases highlight in Fig 4. The cooling load, *P*_*c*_, is based on a 1°C temperature reduction for the flow rates given in the left column. Total power loss is given by: *P*_*T*_ = *P*_*f*_ + *P*_*m*_ + *P*_*l*_. Mmtm—Momentum. Two examples of the 2 m^3^ s^−1^ case are given to illustrate the impact of number of pipes.

Case	Pipe flow	Friction loss	Mmtm loss	Lift loss	Total loss	Cooling rate
*V* / *n*	U (m s^−1^)	*P*_*f*_ (kW)	*P*_*m*_ (kW)	*P*_*l*_ (kW)	*P*_*T*_ (kW)	*P*_*c*_ (kW)
2 m^3^ s^−1^ / 1	2.55	466	7	1	474	8581
2 m^3^ s^−1^ / 2	1.27	117	2	1	120	8581
5 m^3^ s^−1^ / 4	1.59	457	6	2	465	21453
10 m^3^ s^−1^/ 8	1.59	913	12	3	928	42906

Energy calculations (Table 2) show that friction loss dominates over the energy required to accelerate the flow or to lift the water. But pumping cool water to the site, even including a pumping inefficiency (see below), requires far less energy than cooling the water at the site (Table 2, right column).

To cost the energy required, we assume that 1 kWh costs $A 1. This is an expensive rate, but realistic at a remote offshore site requiring peak demand for only a few months of the year. For comparison, in 2019 the annual volume-weighted average spot price in the State of Queensland energy market was $A 0.065 per kWh (Australian Energy Regulator website) and the cost of energy from photovoltaic systems was $A 0.1 per kWh. We assume a pump efficiency of 80%, and an energy cost of 465 kW (sum of friction, momentum and lift loss, row 3 of Table 2). Thus the cost per site per day of the 5 *m*^3^ *s*^−1^ case with four 1 m diameter pipes is 465 kW × 24 h / 0.80 = $A 13,950. For 4 sites the cost is $A 55,800. In this case, 39 + 25 + 22 + 9 + 2 = 97 ha (of the 1334 ha) of Lizard Island reef flat (Fig 4e, sum of histogram column 0.15—0.25 with those to its right) achieved a reduction in temperature of 0.15°C or more. Applied over a 3 month summer, this results in a reduction in Degree Heating Weeks (DHWs) of 0.15 × 90/7 = 1.93 DHWs. Thus to reduce 97 ha of Lizard Island reef flat by ∼ 2 DHWs costs = $A 13,950 × 90 × 4, or around $A 5 M in energy alone.

In this study we restricted our analysis to the power component of the operating costs of cool-water injection, although other aspects of an engineering project such as capital cost, regulatory approvals etc. can be expensive. As an indication, using data from marine outfall pipes used for offshore wastewater discharge [32], a capital cost of $A 5,000 per metre of a single 1 m diameter pipe laid in the marine environment results in a cost of $A 15M for a 3 km long pipe. Without further refining these engineering calculations it is clear that capital costs would an important component of any further optimisation of the project costs, and that moving cool-water over long distances in pipes in the marine environment quickly reduces the feasibility of the intervention.

### Habitat footprint of cool-water injection

Initially we chose Lizard Island among reefs with a long reef age due its reef flat containing more corals than, for example, the broad reef flats of Cockburn Reef. Benthic composition maps from the Allen Coral Atlas [33] show that regions of Lizard Island dominated by coral/algae are principally on the outer slopes of the reef, and the inner reef/lagoon area primarily consists of rock, rubble and sand. As mentioned earlier, the regions of greatest ecological value are typically the exposed reef slopes with fast moving currents that make them less suitable for thermal mitigation through cool-water injections. In the case of Lizard Island, the most effective sites for thermal stress mitigation were the two sites on the southern coastal line of the main island (Fig 4). However, the smaller footprints of the southern and northern most injection sites include areas with greater fractions of coral/algal cover [33]. Further modelling of cool-water injection should involve optimisation the value of the ecosystems protected.

### Limitations of the modelling approach

It would generally be expected that the regional models will have correctly ordered the most feasible reefs, and the unstructured model will have accurately reproduced the changes in temperature on the reef due to the injection. The energy calculations are less certain, and may be much aided by further engineering design, but nonetheless provide reasonable estimates of energy costs.

Much greater uncertainty lies in the effect on the coral communities themselves. Among those factors of concern include: (1) upwelled water will be higher in both nutrients and dissolved inorganic carbon than the surface water it is displacing [34, 35], potentially increasing algal growth and acidifying the waters and; (2) by reducing selection pressure for bleaching mortality, the cool-water injections may inadvertently leave future populations more vulnerable to bleaching mortality than they otherwise would be [36]. While it is beyond the scope of this paper to consider negative ecological impacts to environmental changes (damage to sealife caused by the pipes and pumps, polluting the reef soundscape and the risk of pipe movement during tropical cyclones to name a few), we recognise these will be important in any complete study to optimise cool-water injections.

## Conclusion

In the absence of returning atmospheric carbon dioxide concentrations to those compatible with ocean temperatures below the mass coral bleaching temperature thresholds, the most straightforward means to reduce thermal-stress induced bleaching is to cool the water at the seabed above coral communities. The feasibility of reducing the seabed temperature through local interventions is considered first by analysing the feasibility of doing so on 19 reefs with differing physical environments using both residence time metrics. We then concentrate on Lizard Island, the most promising candidate of the 19 reefs, and develop a 40 m resolution hydrodynamic model to investigate the effect of the injection of cool water at differing volumetric rates. Injecting 27°C at a rate of 5 m^3^ s^−1^ at 4 sites cooled 97 ha of the reef by 0.15°C or more. As a first estimate of costs of injecting cool water on Lizard Island, we calculate the energy costs required for a number of simple pipe configurations accessing water from a nearby, 40 m deep, channel. The energy costs alone of cooling 97 ha of seabed surrounding Lizard Island in summer by ∼2 DHW is $A 5 M. A more precise costing will require further expert engineering design of the pumping equipment. As a result of generally less favourable hydrodynamic properties, cooling seabed temperatures on most of the GBR reefs will be more expensive per hectare than at Lizard Island.

The process of mapping out the feasibility of an intervention across a large number of reefs, identifying a best candidate, and then simulating the effect of the intervention at a particular site is a critical process in scoping out the best initial investments in reef adaptation / restoration. For the case of cool-water injection, this study shows that even for the most favourable reefs, cool-water injection is energy expensive and therefore only possible for a very limited number of high value reefs. Cool-water injection could not be scaled up to any meaningful fraction of the 3,100 reefs of the GBR.

Above all else, this study demonstrates that feasibility of cool-water interventions can vary by many orders of magnitude due to local environmental differences. This variability in feasibility with reef site is likely to be true of other types of reef-scale interventions such as releasing reflective surface films or assisted migration of heat-tolerant corals [6]. In order to select, and then implement, successful interventions, it is critical that an optimisation process is undertaken so that potentially successful interventions are not ignored because they were trialled on non-optimal reefs, interventions that could never be successful are never attempted, and that the limited resources available for interventions are deployed in the most effective circumstances.

## Supporting information

S1 Fig Animation of reef age on 19 reefs of the GBR. Reef age (colour) of the surface water on 19 reefs of the GBR with surface currents shown with grey quivers. To account for different sizes, the smallest reefs are zoomed by twice the largest reefs. The moon phase is shown schematically in the right bottom to emphasise the dependence of reef age on the spring-neap cycle, along with the date and time of the image. The solid and dashed white lines are the 10 and 20 m depth contours. The length of the quiver is the distance water moves in 60 minutes.
(GIF)
