# Supplementary figures and images for: Optimising cool-water injections to reduce thermal stress on coral reefs of the Great Barrier Reef
Source: PLoS One. 2020 Oct 20;15(10):e0239978. doi: 10.1371/journal.pone.0239978 (PMC7575073; doi:10.1371/journal.pone.0239978)

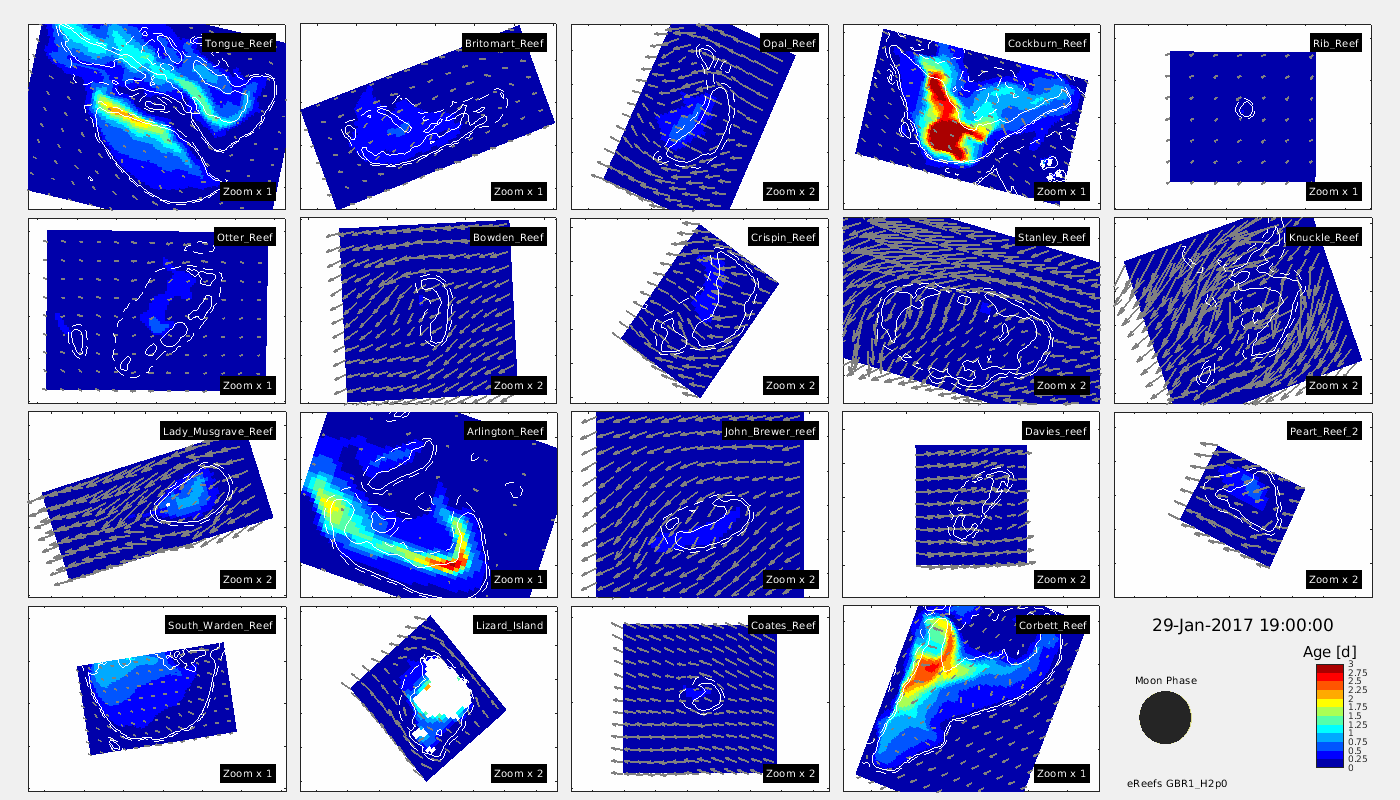

Supplement: S1 Fig — Reef age (colour) of the surface water on 19 reefs of the GBR with surface currents shown with grey quivers. To account for different sizes, the smallest reefs are zoomed by twice the largest reefs. The moon phase is shown schematically in the right bottom to emphasise the dependence of reef age on the spring-neap cycle, along with the date and time of the image. The solid and dashed white lines are the 10 and 20 m depth contours. The length of the quiver is the distance water moves in 60 minutes. (GIF) [file pone.0239978.s001.gif]
